# Epistasis analysis uncovers hidden antibiotic resistance-associated fitness costs hampering the evolution of MRSA

**DOI:** 10.1186/s13059-018-1469-2

**Published:** 2018-07-18

**Authors:** Maho Yokoyama, Emily Stevens, Maisem Laabei, Leann Bacon, Kate Heesom, Sion Bayliss, Nicola Ooi, Alex J. O’Neill, Ewan Murray, Paul Williams, Anneke Lubben, Shaun Reeksting, Guillaume Meric, Ben Pascoe, Samuel K. Sheppard, Mario Recker, Laurence D. Hurst, Ruth C. Massey

**Affiliations:** 1 Milner Centre for Evolution, Department of Biology and Biochemistry, University of Bath, Bath, UK; 2 School of Cellular and Molecular Medicine, University of Bristol, Bristol, UK; 3 Division of Medical Protein Chemistry, Department of Translational Medicine, Lund University, S20502 Malmö, Sweden; 4 University of Bristol Proteomics Facility, University of Bristol, Bristol, UK; 5 Antimicrobial Research Centre, Faculty of Biological Sciences, University of Leeds, Leeds, LS2 9JT UK; 6 Centre for Biomolecular Sciences, School of Life Sciences, University of Nottingham, Nottingham, NG7 2RD UK; 7 Chemical Characterisation and Analysis Facility, Faculty of Science, University of Bath, Bath, BA2 7AY UK; 8 Centre for Mathematics and the Environment, University of Exeter, Penryn Campus, Penryn, TR10 9FE UK

**Keywords:** MRSA, Mupirocin resistance, Fitness costs, GWAS, Epistasis

## Abstract

**Background:**

Fitness costs imposed on bacteria by antibiotic resistance mechanisms are believed to hamper their dissemination. The scale of these costs is highly variable. Some, including resistance of *Staphylococcus aureus* to the clinically important antibiotic mupirocin, have been reported as being cost-free, which suggests that there are few barriers preventing their global spread. However, this is not supported by surveillance data in healthy communities, which indicate that this resistance mechanism is relatively unsuccessful.

**Results:**

Epistasis analysis on two collections of MRSA provides an explanation for this discord, where the mupirocin resistance-conferring mutation of the *ileS* gene appears to affect the levels of toxins produced by *S. aureus* when combined with specific polymorphisms at other loci. Proteomic analysis demonstrates that the activity of the secretory apparatus of the PSM family of toxins is affected by mupirocin resistance. As an energetically costly activity, this reduction in toxicity masks the fitness costs associated with this resistance mutation, a cost that becomes apparent when toxin production becomes necessary. This hidden fitness cost provides a likely explanation for why this mupirocin-resistance mechanism is not more prevalent, given the widespread use of this antibiotic.

**Conclusions:**

With dwindling pools of antibiotics available for use, information on the fitness consequences of the acquisition of resistance may need to be considered when designing antibiotic prescribing policies. However, this study suggests there are levels of depth that we do not understand, and that holistic, surveillance and functional genomics approaches are required to gain this crucial information.

**Electronic supplementary material:**

The online version of this article (10.1186/s13059-018-1469-2) contains supplementary material, which is available to authorized users.

## Background

Antibiotic resistance, which can involve either the mutation of existing genes or the acquisition of genes encoding a resistance mechanism [1], can evolve in many ways and frequently incurs a fitness cost to the organism [2]. As antibiotics are most commonly used for short and defined periods of time, resistant bacteria are under selection to reduce these costs to avoid displacement once treatment has finished. In many cases this is achieved through compensatory mutations that allow many resistance mechanisms to be stably maintained in populations for long periods of time [3]. However, some antibiotic resistance mechanisms have been reported to incur no detectable fitness costs [4–7], which suggests there should be no barriers to their widespread dissemination.

*Staphylococcus aureus* is an example of a major human pathogen [8] that has become more challenging to treat due to the emergence of antibiotic resistance, with methicillin-resistant *S. aureus* (MRSA) being the most notable example [9]. *S. aureus* resides asymptomatically as part of the normal nasal flora of up to 50% of humans [10]; however, this carriage is a significant risk factor for infection [11] to the extent that carriers are often decolonised using antibiotics such as mupirocin prior to invasive procedures, including surgery and dialysis [12]. Mupirocin is a polyketide antibiotic that is applied as an ointment to eradicate nasal carriage of MRSA in patients at risk of infection [13]. Such decolonisation has been reported to reduce *S. aureus* infections of post-surgical wounds by 58%, of haemodialysis patients by 80% and of peritoneal dialysis patients by 63% [14].

The molecular target for mupirocin is the bacterial isoleucyl-tRNA synthetase (IleRS), which charges tRNAs with the amino acid isoleucine (Ile) [13]. By binding to this enzyme the antibiotic halts protein synthesis, and in doing so inhibits bacterial growth [15]. As a consequence of the widespread use of mupirocin, resistance has emerged where the bacteria have mutated the gene encoding IleRS, *ileS*, resulting in an amino acid substitution (e.g. V588F, encoded by a G to T single nucleotide polymorphism (SNP) at position 1762 in the *ileS* gene (G1762 T)) that alters the structure of the protein’s active site without removing its functionality, rendering mupirocin less effective [16]. This confers a low to intermediate level of resistance on the antibiotic [17]. Alternatively, the bacteria may acquire an alternative IleRS, encoded by a *mupA* or *mupB* gene, on a plasmid, which confers a higher level of resistance to mupirocin [18, 19].

The prevalence of mupirocin resistance varies widely, with the highest rates associated with patient groups repeatedly exposed to the antibiotic [20, 21]. It has also been shown that in countries where restrictions were put in place limiting the use of this antibiotic, such as New Zealand and Australia, the prevalence of resistant strains significantly declined [21]. This suggests that antibiotic exposure is required to maintain selection of this resistance mechanism within a population, despite it being reported as incurring no fitness cost.

The *ileS* gene is highly conserved across the thousands of sequenced *S. aureus* isolates, and many failed attempts to inactivate it suggest its activity is essential to the bacteria. It is therefore surprising that the mutation that confers mupirocin resistance, by altering the structure of the encoded protein, does not appear to affect fitness [4]. Particularly given that the replacement of valine 588 with phenylalanine (V588F), a much bulkier residue, is likely to fill and distort the Rossman fold of the enzyme, which is responsible for its ATP binding activity [4, 16]. However, in a recent genome-wide association study (GWAS) on the major hospital acquired MRSA clone, ST239, this mutation was significantly associated with differences in the virulence of *S. aureus* isolates [22]. Its effect on toxin secretion, a major aspect of *S. aureus* virulence, was believed to result from epistatic interactions between *ileS* and other polymorphic loci. Upon analysis of a second genetically and geographically distinct collection of clinical isolates of the USA300 lineage, we detected this epistasis signal again. Here we characterise the effect this mutation has on both toxin production and bacterial fitness and in doing so uncover a potential explanation for the lack of success of these resistant strains.

## Results and discussion

### Validation of the epistatic association between mupirocin resistance and toxicity

**Fig. 1:**
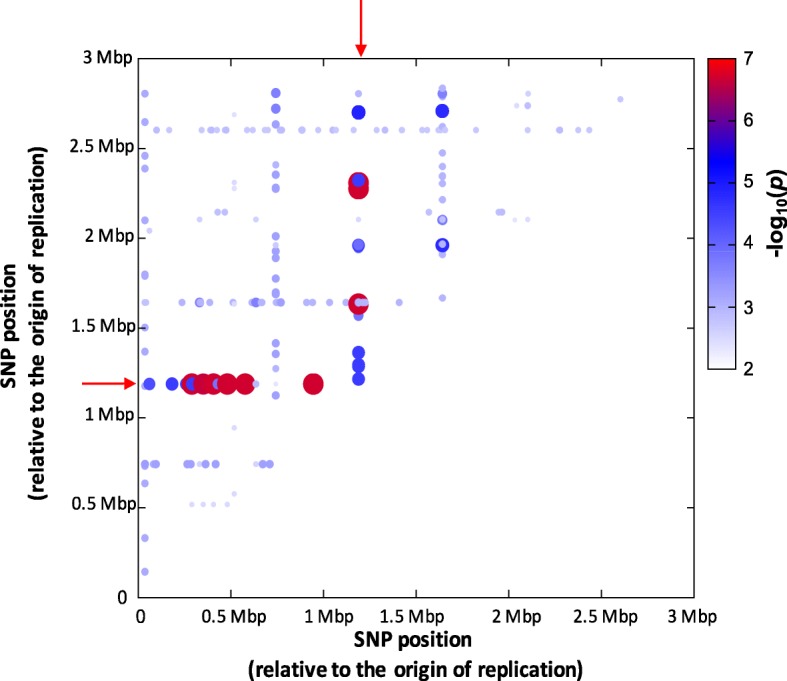
Epistasis between the mupirocin resistance-encoding mutation in the *ileS* gene and many other loci is associated with the toxicity of the USA300 lineage of MRSA. This heat map illustrates where specific combinations of the polymorphic site in the *ileS* gene and polymorphic sites elsewhere on the chromosome are associated with the toxicity of individual isolates. The mup^R^-conferring site is indicated on the x- and y-axes by the *red arrows*

We have previously identified toxicity-associated, epistatic interactions occurring between the mupirocin resistance (mup^R^)-conferring SNP in the *ileS* gene (G1762 T, which confers the V588F change in the protein) and other polymorphic loci within a collection of ST239 MRSA isolates [22]. As the gold standard for validating any GWAS result is to demonstrate the same effect in a subsequent independent population, we analysed the toxicity and sequence data for a collection of 130 USA300 (ST8) MRSA isolates [23]. The epistasis test within the whole genome association analysis toolset PLINK [24] compares the toxicity of isolates with each allele of a specific locus (i.e. those with and without a SNP) in combination with each allele identified at all other loci within the collection, i.e. it tests whether the co-occurrence of SNPs in any two loci is significantly associated with a phenotype. With this approach we found that within this second genetically and geographically distinct collection of clinical isolates the SNP conferring mup^R^ resistance emerged again as the most dominant toxicity-affecting epistatically interacting locus (Fig. 1), verifying our initial results with the ST239 MRSA collection.

### The epistatic signal between the mup^R^ mutation and other loci appears to be indirect

Epistatic signals can emerge from population-based GWAS analyses for several reasons. It might signal that direct interactions are occurring between two loci, such that together they affect a phenotype, for example a transcriptional regulator and the region of DNA it binds to. SNPs can arise in the DNA binding region that affect how the regulator binds to it, or the regulator might acquire SNPs that affect its ability to bind the DNA. As these loci evolve and mutate an epistatic signal associated with the polymorphisms and the phenotype they affect could emerge. Alternatively, the epistatic effect may be a less direct lineage-specific effect, where a polymorphism can have an effect on a phenotype in one genetic background but not in another. In this case the polymorphisms that are associated epistatically with the primary SNP of interest may simply reflect the isolate’s genetic background. To examine whether the mup^R^-conferring SNP has a direct or indirect epistatic effect, we compared the list of loci it was epistatically paired with from the ST239 and the USA300 collections (Additional file 1: Table S1). Other than the mup^R^-conferring SNP, no other loci were in common between the two collections, suggesting that the epistatic effect may be indirect.

To examine whether the mup^R^-conferring epistatic effect was dependent upon a strain’s genetic background, we introduced it into three distinct *S. aureus* strains that all belonged to the ST8 clonal background (as determined by MLST: SH1000, RN6390B and USFL34). The presence of the V588F-conferring mutation and the mup^R^ phenotype were confirmed; however, it was only in the SH1000 background that any effect on toxicity was observed, where the mupirocin-resistant strain, MY40, lysed only 71% of the cells (cultured THP-1 cells) compared to the wild-type mupirocin-sensitive SH1000 strain, which lysed 87% of the cells (*n* = 6, two-tailed *t*-test; *p* = 0.023; the effect of this mutation was further confirmed in four more independently selected mupirocin-resistant isolates of the SH1000 background (Additional file 1: Figure S1). By comparison, this mup^R^ mutation had no effect on toxicity in either the RN6390B or USFL34 backgrounds (*n* = 6; two-tailed t-test *p* = 0.59 and 0.12, respectively). Together these data suggest that the mup^R^-associated epistatic effect observed here is likely to be indirect and its effect on toxicity limited to specific genetic backgrounds.

### The effect of mup^R^ on toxicity is independent of the Agr quorum sensing system

Given the effect on toxicity the mup^R^-conferring SNP has in the SH1000 *S. aureus* strain, we quantified the levels of production of some of the major cytolytic toxins produced by *S. aureus*: alpha toxin and the phenol soluble modulins (PSMs). For this, we first confirmed that no other toxicity-affecting mutations had arisen during our selection of the MY40 (SH1000 mup^R^) strain by comparing the genome sequences of the SH1000 and MY40 strains. The only non-synonymous SNP difference found between these strains was that conferring the V588F change in IleRS, although a small number of synonomous SNP differences were also detected (Additional file 1: Table S2).

**Fig. 2:**
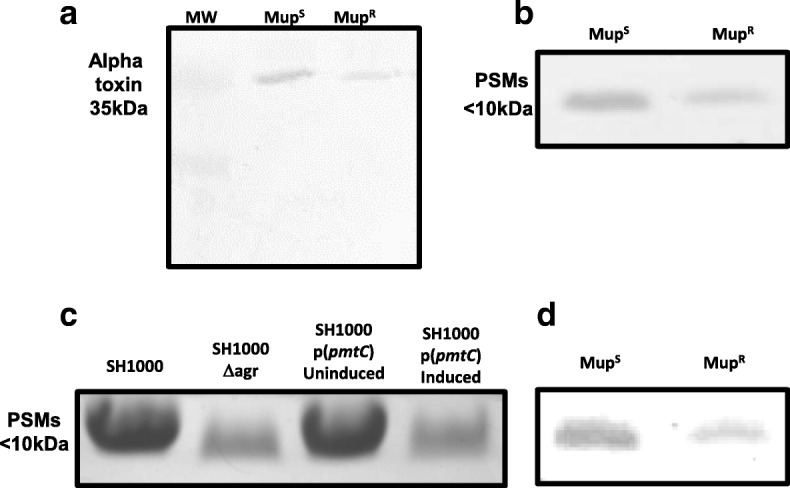
The mup^R^ mutation reduces toxin production by the SH1000 strain. **a** A representative image of a western blot on TCA precipitated bacterial supernatant using anti-alpha toxin antibodies. On average we found the wild-type mup^S^ strain produced twofold more alpha toxin compared to the mup^R^ strain. **b** Coomassie stained SDS-PAGE gel with butanol extractions of bacterial supernatant, containing the PSMs. On average we found the wild-type mup^S^ strain produced 3.4-fold more PSM compared to the mup^R^ strain. **c** Over-expression of the *pmtC* gene, which encodes one of the ATP binding proteins of the PSM secretory system, Pmt, in the wild-type SH1000 strain causes a reduction in the abundance of the PSM in the *S. aureus* supernatant. An Agr mutant has been provided as a control. **d** The intra- and extra-cellular levels of PSMs were pooled to compare the overall levels of PSM production where on average the wild-type strain produced 3.2-fold more PSMs. A full length SDS-PAGE gel is provided in Additional file 1: Figure S3 to illustrate why we only provide a ‘letter-box’ snap-shot of the gels here

To quantify alpha toxin production, the bacteria were grown for 18 h and the proteins in supernatant precipitated using trichloroacetic acid, followed by western blotting using anti-alpha toxin antibodies. For PSM quantification butanol was used to extract the PSMs from the bacterial supernatant, which were then run on SDS-PAGE gels. We found that the wild-type mup^s^ strain SH1000 produced, on average, twofold more alpha toxin and 3.4-fold more PSMs compared to the mup^R^ mutant strain (*n* = 6, two-tailed t-test, *p* = 0.02 and 0.001, respectively; a representative image is presented in Fig. 2a), which explains the results of the THP-1 lysis assay.

Given the dominant effect the Agr quorum regulatory system has on toxin production, we hypothesised that its activity may be affected in the mup^R^ mutant. To test this we quantified RNAIII expression levels, an effector molecule of the Agr system; no difference in expression was detected (*n* = 6, two-tailed *t*-test, *p* = 0.49). We also quantified the response of the mup^R^ and mup^S^ strains to exogenous AIP, the autoinducing pheromone of the Agr system, and we quantified how much AIP the mup^R^ and mup^S^ strains produced. However, we could detect no difference in Agr activity between theses strains by any of these assays (Additional file 1: Figure S2), suggesting that the effect of mup^R^ on toxicity is independent of the Agr system.

### Mupirocin resistance exerts a pleiotropic effect on the *S. aureus* proteome

The protein synthesis-disrupting effect of mupirocin has been used to induce and study the stringent response in bacteria [25]. Exposure to mupirocin has also been shown to affect the expression of enzymes involved in the biosynthesis of other branched chain amino acids (BCAA) and the CodY virulence regulon [26]. As the activity of the Agr system does not appear to be affected by mup^R^, we hypothesised that this mutation may have a similar effect on *S. aureus* that exposure to mupirocin has, which might explain its effect on toxicity. To examine this we adopted a proteomic approach using tandem-mass-tagging (TMT) protein mass spectroscopy [27] on whole cell lysates. As toxin production by *S. aureus* is growth dependent, we harvested proteins during the stationary phase of growth, and where the effect of exposure to mupirocin was being sought, we used a concentration of mupirocin that was below the minimum inhibitory concentration (MIC) to avoid any confounding effects of growth repression. The proteins were therefore extracted from triplicate 18-h cultures in tryptone soy broth (TSB), where we used a concentration of 10 ng/ml mupirocin, which we found to be the highest concentrations of mupirocin for which we found no inhibition of growth of the mup^S^ SH1000 strain.

Of the 3026 open reading frames predicted for the NCTC8325 chromosome (which is the closest reference genome to SH1000), we detected and quantified 1284 proteins, and we used a cut-off of a minimum of a 50% difference in protein abundance to identify differentially produced proteins. When we compared the proteomes of SH1000 and MY40, 135 proteins were differentially produced (Additional file 2: Table S3), suggesting that this resistance mutation has pleiotropic effects on protein production. Exposure of the wild-type SH1000 strain to mupirocin resulted in differential production of 60 proteins when compared to the untreated SH1000 (Additional file 3: Table S4), but the production of only eight proteins was affected both by mupirocin exposure and in the mup^R^ mutant (indicated in Additional file 2: Table S3 and Additional file 3: Table S4), suggesting that while there are some common downstream effects of interfering with the activity of IleRS through either mutation or antibiotic exposure, there are many differences in the outcome.

Interestingly, for both the mup^R^ mutation and exposure to mupirocin we detected an upregulation of IleRS production, suggesting that the bacteria may be able to partially compensate for the interference in the activity of this enzyme by upregulating its production. In relation to previously reported effects on BCAA enzyme production, induction of the stringent response and effects on the CodY virulence regulon, the only effect we observed here was on BCAA enzyme production upon exposure to mupirocin (Additional file 3: Table S4). We believe the differences we report here compared to these previous studies [25, 26] are due to our use of sub-inhibitory concentrations of mupirocin and extracting proteins after 18 h, compared to the methodology used in the other studies where 3× and 5× MICs of mupirocin were used and the protein extracted within an hour of this exposure.

Although we detected no effect on the CodY regulon we did observe a difference between the wild type and mup^R^ mutant in the abundance of the cytoplasmic response regulator of the Agr toxicity regulating system, AgrA, where the mup^R^ mutant produced, on average, sevenfold higher levels of this protein (Additional file 2: Table S3). The *agrA* and *agrC* genes are co-transcribed, but there was no difference in AgrC protein abundance across the protein preparations, which suggests that the effect on AgrA abundance must be post-transcriptional. The AgrA protein needs to be phosphorylated to become transcriptionally active, and as such we would expect to see increased transcription of the RNAIII effector molecule of the Agr system were it active. As mentioned above, however, we detected no difference in RNAIII expression, which suggests that although AgrA may be more abundant in the mutant, it does not seem to be transcriptionally active.

### Mupirocin resistance affects the activity of the PSM secretory apparatus, Pmt

Of the toxins encoded on the SH1000 genome, there was significantly more of both delta toxin and PSMα1 in the lysate of the mupirocin-resistant mutant (35- and 21-fold respectively; Additional file 2: Table S3). These are two of the most abundantly produced members of the PSM family of cytolytic toxins [28]. This result was surprising given that our quantification of the levels of the PSMs in supernatant demonstrated a significantly lower production by the mup^R^ mutant compared to the wild-type strain.

As the PSMs are more abundant in the intracellular environment of the mup^R^ mutant, but less abundant in the extracellular environment, we hypothesised that PSM secretion may be affected in the mutant. This is facilitated by the activity of the PSM export system, Pmt [29], and although the *pmt* genes have not been annotated on the NCTC8325 genome, they are present (locus tags SAOUHSC_02155, SAOUHSC_02154, SAOUHSC_02153, SAOUHSC_02152, SAOUHSC_02151). This locus encodes a regulatory protein, two membrane bound ABC transporter proteins and two ATP binding proteins that fuel the export system, where the four structural genes are transcribed from a single promoter. Each ATP binding protein interacts with its paired transporter protein and the maintained selection of the 1:1:1:1 stoichiometry suggests it is critical to the exporters activity. In our mup^R^ mutant we found that there was more than twice as much of one of the two ATP binding proteins (SAOUHSC_02152, gene name *pmtC*) compared with the wild-type strain. We hypothesised that interference with the stoichiometry of the proteins in this export system may affect its activity, which might explain why we find more PSM inside the cell but less outside. To test this we cloned and expressed the *pmtC* gene, from an inducible promoter in the SH1000 wild-type background, and found that when this ATPase was overexpressed it secreted significantly less PSMs into the extracellular environment (2.8-fold difference, *n* = 6, two-tailed *t*-test, *p* = 0.003; a representative image of this is presented in Fig. 2c). This suggests that the effect mupirocin resistance has on *S. aureus* toxicity is at least partially mediated by interfering with the activity of the PSM secretory apparatus.

It is interesting to consider that the complete inactivation of the Pmt system has been shown to be lethal to the bacteria, presumably as a result of the damage the PSMs can cause to internal membrane structures [29]. Here we demonstrate a partial blocking of the Pmt system by mupirocin resistance, and some accumulation of PSMs internally; however, as we see no in vitro growth defects associated with this it must be a sub-toxic level.

### Reducing the production of toxins alleviates the fitness cost of mup^R^

Toxin production by bacteria is an energetically costly activity [23, 30, 31]. Although we see a significant reduction in alpha toxin production by the mup^R^ mutant, for the PSMs we see an increase in intracellular abundance but a decrease in the extracellular abundance. This makes it difficult to speculate on what fitness consequences there might be as a result of these difference. To clarify this, we compared the total amount (both intra- and extracellular) of PSMs being produced by the wild-type and mup^R^ mutant by pooling whole cell lysates with supernatant prior to performing butanol extractions. Despite there being more PSMs intracellularly in the mutant, this represents such a small proportion of the total amount of PSMs produced by the bacteria that it had no effect on the relative abundance of the PSMs (Fig. 2d), demonstrating that the mup^R^ mutant does produce significantly less of two of the major toxin groups produced by *S. aureus*.

**Fig. 3:**
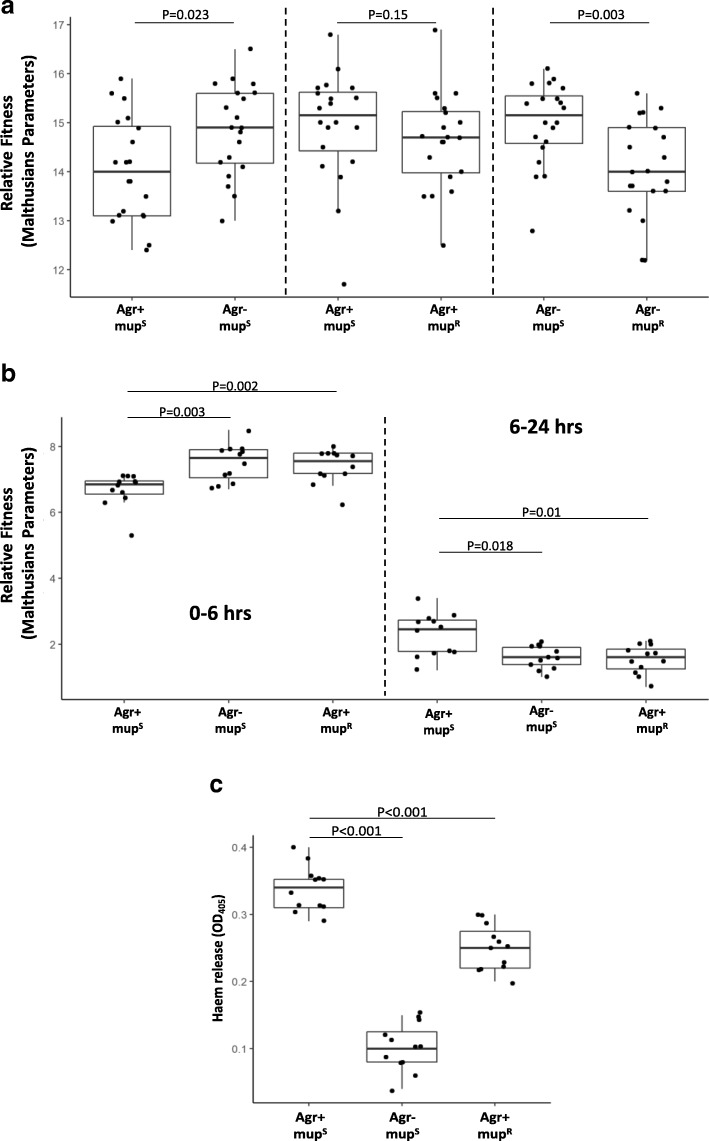
Mupirocin resistance affects the relative fitness of *S. aureus.* **a** The effect of mup^R^ on the relative fitness of *S. aureus* was determined in strains with and without a functioning Agr system by direct competition in TSB. There was no difference in fitness in the Agr+ background, but in the absence of Agr, the mup^R^ strain was less fit. Control competition of an Agr+ vs an Agr− strain is shown. **b** The effect of mupR on relative fitness was determined by individual culture in a nutrient-poor environment (0.1× TSB) supplemented with 5% horse blood. At the early stages of growth (0–6 h) the mup^S^ strain was more fit than both the Agr− and mup^R^ strains. Whereas between 6 and 24 h when the nutrients in the TSB were depleted and cell lysis became necessary, the mup^S^ strains were relatively more fit than both the Agr− and mup^R^ strains. **c** After 24 h of growth in the 0.1× TSB + 5% horse blood medium the Agr+ strain releases more haem into the supernatant relative to the Agr− and mup^R^ strains

The mup^R^ mutation has previously been reported as having no fitness costs, despite occurring in the active region of a highly conserved and essential gene [4, 16]. Here we demonstrate that it has pleiotropic effects on the *S. aureus* proteome. As fitness costs associated with antibiotic resistance can be offset in many ways by bacteria, we hypothesised that perhaps there is a cost to becoming mup^R^, but the associated reduction in the production of toxins compensates for this. To test this we performed a series of competition assays to compare the relative fitness [32] of the mup^S^ and mup^R^ strains in two genetic backgrounds. As a control we first verified that reducing toxin production does make a strain relatively more fit when grown in vitro by competing the mupirocin-sensitive SH1000 against its isogenic Agr mutant, where we found the mutant to be significantly more fit (Fig. 3a; *n* = 20, Mann-Whitney U test, *p* = 0.023). As reported previously, when we competed the mup^S^ wild strain against the mup^R^ strain we observed no difference in fitness (Fig. 3a; *n* = 20, Mann-Whitney U test, *p* = 0.15). However, when we quantified the relative fitness in an Agr defective background where neither strain could produce toxins, such that any alleviation of fitness costs that result from the relative reduction in toxicity of the mup^R^ mutant was nullified, we found the fitness of the mup^R^ MY41 strain to be significantly lower than that of the mup^S^ MY18 strain (Fig. 3a; *n* = 20, Mann-Whitney U test, *p* = 0.003). This demonstrates that this mupirocin resistance-conferring mutation does incur a fitness cost, but that this cost can be masked in vitro by reducing the costly production of toxins.

As the ability to produce toxins is selected for in environments such as the skin and nose [23, 33], we hypothesised that, in such an environment, the costs associated with this resistance mechanism should become apparent. To test this we developed a growth medium with very low levels of nutrients (0.1× TSB) but added 5% horse blood such that strains that can produce toxins can release and utilise further nutrients from these blood cells for growth. To avoid the less toxic cells benefiting from nutrients released by the more toxic bacteria we grew the wild-type mup^S^ SH1000 strain, an Agr mutant in the SH1000 background as a control, and MY40, the SH1000 mup^R^ mutant, separately and quantified their relative fitness (Malthusians parameters) at 3, 6 and 24 h in this medium. After 3 h of incubation in the medium there was no detectable growth of any of the strains; however, by 6 h all three strains had grown, although the Agr mutant and the mup^R^ strain grew faster than the SH1000 strain (Fig. 3b; *n* = 12, Mann-Whitney U test, *p* = 0.003 for the Agr mutant and 0.002 for the mup^R^ mutant). This suggests that the bacteria enter a long lag phase in this medium, but once adapted (after 3 h) sufficient nutrients are available to sustain some growth. At this time point the SH1000 strain is at an apparent disadvantage, possibly by expending energy on producing more toxins than the Agr and mup^R^ mutants, although we would not typically expect *S. aureus* to produce toxins this early in its growth phase.

After the 6-h time point there was a distinct shift in the relative fitness of the strains, with the SH1000 strain growing faster than the Agr mutant and the mup^R^ strain (Fig. 3b; n = 12, Mann-Whitney U test, *p* = 0.018 and 0.01, respectively). The increased relative growth rate of the wild-type SH1000 strain could be explained by its increased capability to lyse cells and release nutrients necessary for its growth. To verify this, at the end of the growth assay we removed the bacteria and intact blood cells by centrifugation, and quantified by spectroscopy the levels of haem released from the cells. Significantly more haem was present in the supernatant of the SH1000 strain (Fig. 3c; *n* = 12, Mann-Whitney U test, *p* < 0.001 in both cases), demonstrating that SH1000 has released more cellular material into the broth.

While the relative fitness of any organism is dependent upon its environment, here we demonstrate how readily this can fluctuate within an environment. That the fitness consequences of mupirocin resistance became apparent when cell lysis became necessary may provide an explanation for why this resistance-conferring mutation, despite the ease with which it can emerge, is not more prevalent and is readily lost within healthy communities [21].

## Conclusions

Understanding the evolution of antibiotic resistance, in terms of both how it emerges and how it is stably maintained, is critical if ways to curtail its further emergence are to be developed. With some antibiotic resistance mechanisms being reported as incurring no fitness costs [4–6], and others where physiological and regulatory compensation of costs has been demonstrated [23, 31, 33–35], it is perhaps surprising that they have not become more prevalent. Here, by adopting a population-based functional genomics approach, we have uncovered hidden fitness costs associated with an apparently cost-free, antibiotic resistance mechanism. We have demonstrated that the mupirocin resistance-conferring mutation of the gene encoding the IleRS enzyme has pleiotropic effects on bacteria, which in hindsight is perhaps unsurprising given the highly conserved and essential nature of this gene. In this instance, while reducing the energetic expense associated with toxin production decreases the resistance-associated fitness costs, this appears to be unsustainable when the ability to produce toxins is required. Given that the nose of healthy carriers has been shown to be such a toxicity-favouring environment for *S. aureus*, this toxicity-related fitness off-setting may explain why this resistance mechanism is not more prevalent, and is readily displaced, once this antibiotic has been removed from their environment. An effect we would have been unable to explain had we not adopted a population-based approach to studying this major bacterial pathogen.

## Methods

### Strains and growth conditions

All strains used in this study are listed in Additional file 1: Table S5. *S. aureus* strains were grown at 37 °C, in either tryptic soy agar or broth (TSA/TSB) with the appropriate antibiotic where necessary. The *Escherichia coli* TOP10 strain containing the pAgrC(his)A plasmid was grown in Luria-Bertani (LB) media with 100 μg/ml ampicillin. THP-1 cells were grown in RPMI 1640 supplemented with foetal bovine serum (10%), L-glutamine (2 mM), penicillin (100 units/ml) and streptomycin (100 μg/ml) and incubated at 37 °C with 5% CO_2_.

### Epistasis analysis

Analysis of SNP–SNP epistatic interactions was performed using the “epistasis” option in the PLINK software package (http://zzz.bwh.harvard.edu/plink/) [24], which is based on linear regression analysis and tests the inclusion of an interaction term (into the regression equation) for statistical significance. The total number of non-synonomous SNPs across our collection of 130 USA300 isolates was just under 4000; however, only those with a minor allele frequency of > 3% were considered, and as interactions between two SNPs can only be tested where both coincide, the number of tests per locus was on average just over 100.

### Selection of the mupirocin resistant strain

As an essential gene *ileS* cannot be inactivated and, as a consequence, a homologous recombination based mutational approach was unsuccessful. Therefore, to generate isogenic mupirocin-resistant and -sensitive strains we utilised a selection-based method where 10^8^ bacteria (100 μl of an overnight culture) on mupirocin containing TSA plates (4 μg/ml) and on average between two and ten mupirocin-resistant colonies emerged on each plate after 48 h incubation at 37 °C. Resistant colonies that emerged were isolated by streaking on fresh antibiotic-containing plates and mutants with the V588F-conferring mutations identified by the following mismatch PCR approach. Allele specific PCR was performed to determine what nucleotide is present at 1762 of the *ileS* gene; G results in V588, which is mupirocin sensitive, and T results in F588, which confers resistance to mupirocin. Each resistant colony was screened with three different primer sets:

1. a control set to confirm the presence of the *ileS* gene: IleS-F-control and IleS-R
2. a set to determine the presence of a G at position 1762: IleS-F-mupS and IleS-R
3. a set to determine the presence of a T at position 1762: IleS-F-mupR and IleS-R

The reactions were set up as recommended for GoTaq (Promega) and the cycling conditions were as recommended by the manufacturer; 50.5 °C was used as the annealing temperature and the extension time was 1 min.

**Table:** 

Primer name	Purpose	Sequence
IleS-F-control	Forward control primer common to both resistant and sensitive bacteria	CTTATAAATTCTTACTTTCTCATGGTTTT
IleS-F-mupS	Forward primer matched to the wild-type mup^S^sequence	TAAATTCTTACTTTCTCATGGTTTTGG
IleS-F-mupR	Forward primer with two mismatches which amplifies the mup^R^ sequence	TAAATTCTTACTTTCTCATGGTTTCT
IleS-R	Reverse primer common to both resistant and sensitive bacteria	GATTGGTGCTAACAACTTCGTCATA

### Genome sequencing of the mup^R^ strain

*S. aureus* strain MY40 was sequenced in this study; DNA was extracted using the QIAamp NA Mini Kit (QIAGEN, Crawley, UK) using the manufacturer’s instructions with 1.5 μg/μL lysostaphin (Ambi Products LLC, NY, USA) to facilitate cell lysis. DNA was quantified using a Nanodrop spectrophotometer, as well as the Quant-iT DNA Assay Kit (Life Technologies, Paisley, UK) before sequencing. High-throughput genome sequencing was performed using a MiSeq machine (Illumina, San Diego, CA, USA) and the short read, paired-end data were assembled using the de novo assembly algorithm SPAdes [36]. Sequence data are archived in the NCBI repositories: GenBank accession NIPL00000000.1, Short Read Archive (SRA) SRR5651527, associated with BioProject PRJNA384009. Assembled genomes are also available on FigShare (doi.org/10.6084/m9.figshare.5089939.v1).

### Toxicity assays

THP-1 cells [37] were grown as described above and harvested by centrifugation and washed in phosphate buffered saline (PBS) and diluted to a final density (determined by haemocytometer) of 2 × 10^6^ cells/ml of PBS. Bacterial supernatant was harvested after 18 h of growth in TSB at 37 °C. The bacterial supernatant (20 μl) was mixed with 20 μl of THP-1 cells and incubated for 12 min at 37 °C. Guava ViaCount (260 μl; Milipore) was added to the sample and incubated at room temperature for 5 min before analysing the viability on the Guava flow cytometer (Milipore). Each strain was assayed six times (three biological replicates and two technical replicates).

### Fitness assays

The strains were cultured individually overnight and diluted to 10^4^ cfu/ml. For the direct competitions 25 μl of each diluted culture was added into 5 ml fresh TSB and grown at 37 °C with shaking for 24 h. The mixed culture was diluted and plated onto agar plates with and without 4 μg/ml mupirocin and incubated at 37 °C. The resulting colonies were counted and the number of colonies from the mupirocin plate was subtracted from the count from no antibiotic plate. The Malthusian parameter was calculated using the following formula:

$$ \mathrm{Ln}\ \left(\mathrm{Final}\ \mathrm{density}\ \left(\mathrm{colony}\ \mathrm{forming}\ \mathrm{units}\ \left(\mathrm{CFU}\right)/\mathrm{ml}\right)/\mathrm{Starting}\ \mathrm{density}\ \left(\mathrm{CFU}/\mathrm{ml}\right)\right) $$

The Malthusian parameters of the mup^S^ and mup^R^ strains were compared using a Mann-Whitney U test as the data were not normally distributed. For the individual fitness assays the strains were cultured overnight, diluted and added to 0.1× TSB (made with PBS rather than water to maintain the integrity of the horse blood cells) to which 5% horse blood (Oxoid) was added. Bacterial growth was determined by plating the cultures on TSA plates after 3, 6 and 24 h of incubation at 37 °C in air. Each competition was performed between 12 and 20, either three biological replicates and three technical or four biological replicates and five technical.

### Deletion of the *agr* locus from SH1000 and MY40

Phage transduction was used to construct the mup^R^ and mup^S^ Agr mutants. In the *S. aureus* strain ROJ48, the entire Agr locus has been replaced with an erythromycin resistance cassette [38]. This was moved from ROJ48 into SH1000 by phage transduction as follows: ROJ48 ϕ11 lysates were prepared from 200 μl of overnight ROJ48 culture in LK (1% tryptone, 0.5% yeast extract, 1.6% KCl) which was added to 3 ml of fresh LK and 3 ml of phage buffer (10 mM MgSO_4_, 4 mM CaCl_2_, 50 mM Tris-HCl pH 7.8, 100 mM NaCl and 0.1% gelatine powder in molecular/MiliQ water), and to this 500 μl ϕ11-RN6390B lysate was added. This was incubated at 30 °C shaking until the media became clear, which indicated bacterial lysis. The lysates were then filter sterilised and a second round of lysis was carried out on ROJ48 with this first round lysate. After these two lysis steps, transduction into SH1000 and MY40 strains was performed by adding 200 μl of overnight culture to 1.8 ml LK with 10 μl 1 M CaCl_2_ and 500 μl the ϕ11-ROJ48 lysate. This was incubated at 37 °C with shaking for 45 min; then 1 ml ice-cold 20 mM trisodium citrate was added and the transducing mixture was placed on ice for 5 min. The bacteria were harvested by centrifugation and re-suspended with 1 ml ice-cold 20 mM trisodium citrate. This was incubated on ice for 2.5 h and plated onto TSA plates with 20 mM trisodium citrate, erythromycin and lincomycin (25 μg/ml), which was incubated overnight at 37 °C.

### qRT-PCR

Overnight cultures were diluted 1:500 into 3 ml fresh TSB-chloramphenicol. After 18 h of growth 2 ml of this culture was mixed with 4 ml RNA Protect Bacteria (Qiagen), and the RNeasy Mini Kit (Qiagen) was used to extract RNA following the manufacturer’s protocol. Lysostaphin (200 μg/ml) was added to Tris-EDTA buffer (Ambion), and this was added to the sample after the RNA Protect step before continuing with the protocol. When the RNA was extracted, a Turbo DNA-free kit (Thermo) was used to remove genomic DNA from the RNA samples; 3 μl Turbo DNase was added to the sample and incubated for 1.5 h at 37 °C, then a further 4 μl Turbo DNase was added and incubated for 1.5 h. DNase inactivation reagent (35 μl) was added to the samples to inactivate the DNase according to the protocol. The concentration of RNA in the samples was measured using a Qubit RNA Broad Range Kit (Thermo) and normalised before using a QuantiTect Reverse Transcription Kit (Qiagen) to convert the RNA samples into cDNA according to the manufacturer’s protocol. After adding the reverse transcriptase, the samples were incubated at 42 °C for 20 min before raising the temperature to 95 °C for 3 min to inactivate the reverse transcriptase. Primers for *gyrB*, a housekeeping gene, were used alongside those for RNAIII to standardise transcript levels (gyrB forward, CCAGGTAAATTAGCCGATTGC; gyrB reverse, AAATCGCCTGCGTTCTAGAG; RNAIII forward, AGCATGTAAGCTATCGTAAACAAC; RNAIII reverse, TTCAATCTATTTTTGGGGATG). ssoAdvanced SYBR Green Supermix (Bio-Rad) was used using a standard curve of known genomic DNA concentrations for each primer set. Samples, standards and water (5 μl) were pipetted into the wells of a 96-well PCR plate. The supermix was added to water and primers according to the manufacturer’s protocol, and 15 μl of this mix was pipetted over the DNA samples. This was then placed into a qPCR machine and run using the manufacturer’s recommendation. The quantity of RNAIII cDNA was divided by the quantity of *gyrB* cDNA to get a ratio of RNAIII transcription levels. This was performed six times, three biological replicates and two technical.

### AIP EC_50_ and AIP quantification assays

These assays were performed as described previously [38]. Strains were grown overnight in Brain Heart Infusion (BHI), and 1 ml of the overnight was washed three times with PBS. The pellet was re-suspended in fresh BHI and grown for a further 2 h. A dilution series of AIP-1 was created, with concentrations ranging from 250 to 0.125 nM in 1:2 dilutions, plus 1250 and 2500 nM. The culture (190 μl) was pipetted into a black 96-well plate, and 10 μl of this dilution series was also pipetted into the wells. The OD_600_ and luminescence readings were carried out every 15 min for 80 cycles in a Tecan plate reader. For the AIP quantification strains were grown in BHI with chloramphenicol (10 μg/ml). The reporter strain was diluted 1/20 into fresh BHI and grown for a futher 2 h, while the supernatants of the mup^S^ and mup^R^ strains were filtered through a 0.22 μm filter. The reporter strain was diluted 1/50 and pipetted into a black 96-well plate. The supernatant was added to the wells at 5% final concentration, and the OD_600_ and luminescence readings were carried out every 15 min for 80 cycles in a Tecan plate reader. This was performed six times, three biological replicates and two technical.

### PSM quantification in supernatants

An overnight culture of SH1000 and MY40 was diluted 1:1000 into 50 ml fresh TSB and grown for 18 h. The cultures were centrifuged at 18,000 rpm for 10 min and 35 ml of the supernatant was mixed with 10 ml butanol. The samples were incubated at 37 °C shaking for 3 h and then centrifuged at 3000 rpm for 3 min; 1 ml of the upper organic layer was taken off. The samples were then freeze-dried overnight and then re-suspended in 160 μl 8 M urea, separated on an SDS-PAGE gel and PSMs quantified by densitometry analysis using the ImageJ software. This was performed six times, three biological replicates and two technical.

### TMT labelling and high pH reversed-phase chromatography

Aliquots of 100 μg of up to ten samples per experiment were digested with trypsin (2.5 μg trypsin per 100 μg protein; 37 °C, overnight), labelled with Tandem Mass Tag (TMT) ten plex reagents according to the manufacturer’s protocol (Thermo Fisher Scientific) and the labelled samples pooled. An aliquot of the pooled sample was evaporated to dryness and resuspended in buffer A (20 mM ammonium hydroxide, pH 10) prior to fractionation by high pH reversed-phase chromatography using an Ultimate 3000 liquid chromatography system (Thermo Fisher Scientific). In brief, the sample was loaded onto an XBridge BEH C18 column (130 Å, 3.5 μm, 2.1 mm × 150 mm, Waters, UK) in buffer A and peptides eluted with an increasing gradient of buffer B (20 mM ammonium hydroxide in acetonitrile, pH 10) from 0 to 95% over 60 min. The resulting fractions were evaporated to dryness and resuspended in 1% formic acid prior to analysis by nano-LC MSMS using an Orbitrap Fusion Tribrid mass spectrometer (Thermo Scientific).

### Nano-LC mass spectrometry

High pH RP fractions were further fractionated using an Ultimate 3000 nanoHPLC system in line with an Orbitrap Fusion Tribrid mass spectrometer (Thermo Scientific). In brief, peptides in 1% (vol/vol) formic acid were injected onto an Acclaim PepMap C18 nano-trap column (Thermo Scientific). After washing with 0.5% (vol/vol) acetonitrile 0.1% (vol/vol), formic acid peptides were resolved on a 250 mm × 75 μm Acclaim PepMap C18 reverse phase analytical column (Thermo Scientific) over a 150 min organic gradient, using seven gradient segments (1–6% solvent B over 1 min, 6–15% B over 58 min, 15–32% B over 58 min, 32–40% B over 5 min, 40–90% B over 1 min, held at 90% B for 6 min and then reduced to 1% B over 1 min) with a flow rate of 300 nl min^− 1^. Solvent A was 0.1% formic acid and solvent B was aqueous 80% acetonitrile in 0.1% formic acid. Peptides were ionised by nano-electrospray ionisation at 2.0 kV using a stainless steel emitter with an internal diameter of 30 μm (Thermo Scientific) and a capillary temperature of 275 °C.

All spectra were acquired using an Orbitrap Fusion Tribrid mass spectrometer controlled by Xcalibur 2.0 software (Thermo Scientific) and operated in data-dependent acquisition mode using an SPS-MS3 workflow. FTMS1 spectra were collected at a resolution of 120,000 with an automatic gain control (AGC) target of 200,000 and a max injection time of 50 ms. Precursors were filtered with an intensity threshold of 5000 according to charge state (to include charge states 2–7) and with monoisotopic precursor selection. Previously interrogated precursors were excluded using a dynamic window (60s ± 10 ppm). The MS2 precursors were isolated with a quadrupole mass filter set to a width of 1.2 m/z. ITMS2 spectra were collected with an AGC target of 10,000, max injection time of 70 ms and CID collision energy of 35%.

For FTMS3 analysis, the Orbitrap was operated at 50,000 resolution with an AGC target of 50,000 and a max injection time of 105 ms. Precursors were fragmented by high energy collision dissociation (HCD) at a normalised collision energy of 60% to ensure maximal TMT reporter ion yield. Synchronous precursor selection (SPS) was enabled to include up to five MS2 fragment ions in the FTMS3 scan**.**

### Proteomic data analysis

The raw data files were processed and quantified using Proteome Discoverer software v2.1 (Thermo Scientific) and searched against the UniProt *Staphylococcus aureus* strain NCTC 8325 database using the SEQUEST algorithm [39]. Peptide precursor mass tolerance was set at 10 ppm and MS/MS tolerance was set at 0.6 Da. Search criteria included oxidation of methionine (+ 15.9949) as a variable modification and carbamidomethylation of cysteine (+ 57.0214) and the addition of the TMT mass tag (+ 229.163) to peptide N-termini and lysine as fixed modifications. Searches were performed with full tryptic digestion and a maximum of two missed cleavages were allowed. The reverse database search option was enabled and all peptide data were filtered to satisfy a false discovery rate of 5%.

## Additional files

Additional file 1: **Table S1.** ST239 and USA300 loci in epistasis with mupirocin resistance. **Figure S1.** The mupR mutation reduces the toxicity of the *S. aureus* strain SH1000. **Table S2.** DNA sequence results for SH1000 and MY40. **Figure S2.** Agr activity is not affected by the mupR mutation. **Figure S3.** Full length SDS-PAGE gel with butanol-extracted PSMs. **Table S5.** Strains constructed and used in this study. (DOCX 571 kb)

Additional file 2: **Table S3.** Protein profile differences between SH1000 (mupS) and MY40 (mupR). Proteins highlighted in *yellow* are also differentially produced by exposure to mupirocin. (XLSX 32 kb)

Additional file 3: **Table S4.** Effect of exposure to mupirocin on SH1000 protein profile. Proteins highlighted in *yellow* are those also differentially produced in the mupR mutant. (XLSX 13 kb)
