# Supplementary material for: Epistasis analysis uncovers hidden antibiotic resistance-associated fitness costs hampering the evolution of MRSA
Source: Genome Biol. 2018 Jul 18;19:94. doi: 10.1186/s13059-018-1469-2 (PMC6052701; doi:10.1186/s13059-018-1469-2)
Supplement: Supplementary file 1 — Table S1. ST239 and USA300 loci in epistasis with mupirocin resistance. Figure S1. The mupR mutation reduces the toxicity of the S. aureus strain SH1000. Table S2. DNA sequence results for SH1000 and MY40. Figure S2. Agr activity is not affected by the mupR mutation. Figure S3. Full length SDS-PAGE gel with butanol-extracted PSMs. Table S5. Strains constructed and used in this study. (DOCX 571 kb) [file 13059_2018_1469_MOESM1_ESM.docx]

**Additional file 1**

**Content:**

**Table S1:** ST239 and USA300 loci in epistasis with mupirocin resistance

**Fig. S1:** The mup^R^ mutation reduces the toxicity of the S. aureus strain SH1000.

**Table S2:** DNA sequence results for SH1000 and MY40.

**Fig. S2:** Agr activity is not affected by the mup^R^ mutation.

**Fig. S3:** Full length SDS-PAGE gel with butanol extracted PSMs.

**Table S5:** Strains constructed and used in this study.

**Table S1:** Loci in both the ST239 and USA300 collections identified by GWAS as interacting epistatically with the mutation in *ileS.*

**ST239**

| **SNP position** | **Locus Tag/Gene Name** | **Putative function** |
| --- | --- | --- |
| 1360889 | SAWT20_12700 | Putative DNA translocase (FtsK/SpoIIIE family protein) |
| 1474673 | SATW20_13760 | Haloacid dehalogenase-like hydrolase superfamily protein |
| 1536348 | *ebh* | Very large surface anchored protein |
| 1557020 | *ebh* | Very large surface anchored protein |
| 1557275 | *ebh* | Very large surface anchored protein |
| 1579791 | *pbp2* | Penicillin-binding protein 2 |
| 1937314 | SATW20_17780 | Putative exported protein |
| 1941832 | SATW20_17820 | Conserved hypothetical protein |
| 1975607 | SATW20_18180 | Lantibiotic biosynthesis protein |
| 2033565 | SATW20_18600 | ABC transporter ATP-binding protein |
| 2075672 | *pcrB* | pcrB family protein |
| 2128192 | SATW20_19530 | β converting phage protein |
| 2312226 | SATW20_21880 | ABC transporter ATP-binding protein |
| 2409540 | SATW20_22770 | putative non-haem iron-containing ferritin |
| 2432219 | *fmtB* | LPXTG surface-anchored protein |
| 2450342 | Intergenic between SATW20_r160 AND SATW20_23010 | 16S rRNA AND conserved hypothetical protein |
| 2548323 | *modC* | Putative molybdenum transport ATP-binding protein |
| 2578126 | SATW20_24400 | Putative bifunctional protein |
| 2639747 | *lldP2* | Putative L-lactate permease 2 |
| 2657438 | Intergenic between SATW20_25130 AND *gltT* | Putative exported protein AND putative proton/sodium-glutamate symport protein |
| 2674904 | *nasD* | Nitrite reductase large subunit |
| 2759775 | SATW20_26050 | Putative short chain dehydrogenase |
| 2790429 | SATW20_26280 | Conserved hypothetical protein |
| 2810368 | Intergenic between SATW20_26460 AND SATW20_26470 | Putative haloacid dehalogenase-like hydrolase AND ABC transporter ATP-binding protein |
| 2970902 | SATW20_27860 | Hypothetical protein |
| 3002241 | *hisH* | Putative amidotransferase |
| 3002845 | *hisB* | Putative imidazoleglycerol-phosphatedehydratase |

**Table S1 (continued):** Loci in both the ST239 and USA300 collections identified by GWAS as interacting epistatically with the mutation in *ileS.*

**USA300**

| **SNP position** | **Locus Tag/Gene Name** | **Putative function** |
| --- | --- | --- |
| 61025 | SAUSA300_0050 | Hypothetical protein |
| 182746 | *cap5I* | Capsular polysaccharide biosynthesis protein |
| 260357 | Intergenic between SAUSA300_0219 AND *pflB* | Putative iron compound A C transporter, iron compound-binding protein AND formate acetyltransferase |
| 270510 | SAUSA300_0226 | 3-hydroxyacyl-CoA dehydrogenase |
| 289635 | SAUSA300_0239 | PTS system, fructose-specific enzyme II, BC component |
| 292738 | *gutB* | Sorbitol dehydrogenase |
| 331125 | SAUSA300_0279 | Putative membrane protein |
| 351171 | SAUSA300_0300 | Conserved hypothetical protein |
| 406847 | SAUSA300_0355 | Acetyl-CoA acetyltransferase |
| 429073 | *ahpF* | Alkyl hydroperoxide reductase, subunit F |
| 467549 | SAUSA300_0414 | Staphylococcal tandem lipoprotein |
| 480640 | SAUSA300_0426 | Conserved hypothetical protein |
| 577067 | *cysE* | Serine acetyltransferase |
| 635299 | *vraB* | Acetyl-CoA c-acetyltransferase |
| 742782 | Intergenic between SAUSA300_0669 AND SAUSA300_0670 | Undecaprenol kinase AND ABC transporter, ATP-binding protein, MsbA family |
| 944770 | Intergenic between *argG* AND *pgi* | Argininosuccinate synthase AND glucose-6-phosphate isomerase |
| 1215913 | *sun* | Ribosomal RNA small subunit methyltransferase B |
| 1286986 | *ftsK* | DNA translocase FtsK |
| 1295497 | *cinA* | Competence/damage-inducible protein cinA |
| 1363207 | *sbcC* | Exonuclease SbcC |
| 1568945 | SAUSA300_1403 | phiSLT ORF412-like protein, portal protein |
| 1632033 | Intergenic between SAUSA300_1477 AND SAUSA300_1478 | Transposase AND lipoprotein |
| 1640549 | SAUSA300_1485 | Conserved hypothetical protein |
| 1944161 | Intergenic between *splA* AND SAUSA300_1759 | Serine protease AND hypothetical protein |
| 1961241 | SAUSA300_1778 | tRNA-asp |
| 2102731 | SAUSA300_1934 | phi77 ORF020-like protein, phage major tail protein |
| 2275521 | SAUSA300_2106 | Putative transcriptional repressor |
| 2276564 | *mtlA* | PTS system, mannitol specific IIA component |
| 2308707 | SAUSA300_2135 | Iron compound ABC transporter, permease protein |
| 2322700 | SAUSA300_2146 | Alcohol dehydrogenase, zinc-containing |
| 2685939 | SAUSA300_2486 | Putative ATP-dependent Clp proteinase |
| 2700617 | SAUSA300_2497 | Aminotransferase, class I |
| 2803422 | SAUSA300_2583 | Putative glycosyl transferase |
|  |  |  |

**Fig. S1:** The mup^R^ mutation reduces the toxicity of the *S. aureus* strain SH1000. The toxicity of five independent mup^R^ isolates, each with the V588F conferring SNP were quantified, in each case their ability to lyse THP-1 cells was reduced upon acquisition of this resistance SNP.

**Table S2:** DNA sequence results for SH1000 and MY40. The strains were sequenced and mapped to the closest reference strain NCTC8325. Presented here are the SNP differences between SH1000 and MY40 having filtered out all the differences they had in common with respect to the reference strain. The only non-synonymous change was the mupirocin resistance conferring mutation in the *ileS* gene. (CDS: coding sequences; N/A: not applicable; hypo: hypothetical; Syn: synonymous)

| **SNP position** | **Feature** | **Gene name/locus-tag** | **Nucleotide in SH1000** | **Nucleotide in MY40** | **Amino acid change** |
| --- | --- | --- | --- | --- | --- |
| 75276 | Intergenic | N/A | T | A | N/A |
| 290135 | CDS | SAOUHSC_00270  (hypo. protein) | A | G | Syn |
| 290144 | CDS | SAOUHSC_00270  (hypo. protein) | T | A | Syn |
| 290147 | CDS | SAOUHSC_00270  (hypo. protein) | T | C | Syn |
| 290149 | CDS | SAOUHSC_00270  (hypo. protein) | C | T | Syn |
| 405366 | Intergenic | N/A | G | A | N/A |
| 841103 | CDS | SAOUHSC_00877 (hypo. protein) | G | T | syn |
| 841139 | CDS | SAOUHSC_00877 (hypo. protein) | G | T | Syn |
| \| 1108891 \| \| --- \| | CDS | Isoleucyl-tRNA synthetase | G | T | V588F |
| 1562913 | Intergenic | N/A | T | A | N/A |
| 2134749 | CDS | SAOUHSC_A02189 (hypo. protein) | T | C | Syn |
| 2244467 | Intergenic | N/A | G | A | N/A |
| 2244495 | Intergenic | N/A | A | G | N/A |
| 2661023 | CDS | SAOUHSC_02887  (hypo. protein) | C | T | Syn |

**Fig. S2:** Agr activity is not affected by the mup^R^ mutation. Effect of mupirocin resistance on the response to exogenous AIP, and on AIP production as a function of growth. **A**: The half maximal effective concentration (EC_50_) of AIP required to activate the Agr system was quantified for both the mupirocin resistant and sensitive *S. aureus* strains, where no significant effect of mupirocin resistance was observed. **B**: The relative concentration of AIP in the culture supernatant was quantified following growth for 6h for both the mupirocin resistant and sensitive strains. No effect of mupirocin resistance on this aspect of Agr activity was observed under these conditions.

**Fig. S3:** Full length SDS-Page gel with PSM extractions from SH1000 (mup^S^) and MY40 (mup^R^). MW: molecular weight standards. Due to their small size the PSMs run in front of the loading dye front making quite a messy gel. This has been provided to explain why only a ‘letterbox’ snap-shot of these gels is provided in the main manuscript file.

**Table S5:** Strains constructed and used in this study.

| **Strain** | **Relevant genotypic information** | **Mupirocin resistance** |
| --- | --- | --- |
| SH1000 | wild-type lab strain | mup^S^ |
| MY40 | mupirocin resistant SH1000 | mup^R^ |
| MY46 | SH1000 (pPmtC) | mup^S^ |
| ROJ48 | Strain 8325-4 *agr::erm^R^* | mup^S^ |
| MY18 | SH1000 *agr::erm^R^* | mup^S^ |
| MY41 | MY40 *agr::erm^R^* | mup^R^ |
| RN6390B | wild-type lab strain | mup^S^ |
| USFL34 | Clinical USA300 isolate | mup^S^ |
| MY47 | Mupirocin resistant RN6390B | mup^R^ |
| MY48 | Mupirocin resistant USFL34 | mup^R^ |
